# Beyond Effectiveness—The Adversities of Implementing a Fortification Program. A Case Study on the Quality of Iron Fortification of Fish and Soy Sauce in Cambodia

**DOI:** 10.3390/nu8020094

**Published:** 2016-02-17

**Authors:** Arnaud Laillou, Simon Pfanner, Theary Chan, Chantum Chea, Borath Mam, Pol Sambath, Saphoon Vonthanak, Frank Wieringa

**Affiliations:** 1 United Nations Children’s Fund (UNICEF), No. 11 Street 75, Sangkat Sraschark, Phnom Penh 12100, Cambodia; 2 Reproductive and Child Health Alliance (RACHA), No. 160 Street 71, Tonle Bassac, Chamkar Mon, Phnom Pen 12100, Cambodia; pfannersimon@gmail.com (S.P.); ctheary@racha.org.kh (T.C.); psambath@racha.org.kh (P.S.); 3 National Sub-Committee of Food Fortification, Ministry of Planning, 386 Monivong Blvd, Phnom Penh 12100, Cambodia; chantumch@gmail.com (C.C.); borathmam@yahoo.com (B.M.); 4 National Institute of Public Health, No. 2 Sangkat Boeungkak, Phnom Penh 12100, Cambodia; svonthanak@gmail.com; 5 UMR 204 “Prevention of Malnutrition and Associated Diseases”, IRD-UM2-UM1, Institute of Research for Development (IRD), BP 645, Montpellier cedex 34394, France; franck.wieringa@ird.fr

**Keywords:** Cambodia, fortified sauces, iron, compliance, Codex, quality control

## Abstract

Fortification of fish and soy sauces is a cost-effective strategy to deliver and increase iron intake in the Cambodian diet, as both are widely consumed by the entire population. In order to qualify as fortified sauces recognized by international regulations, iron content must be between 230 and 460 mg/L, whilst nitrogen and salt should contain no less than 10 g/L and 200 g/L respectively. This survey aims to analyze the progress of the fortification program. Through a better understanding of its obstacles and successes, the paper will then consider approaches to strengthen the program. Two hundred and fifty two samples were collected from 186 plants and 66 markets in various provinces. They were then analyzed for iron, nitrogen and salt content. The study demonstrates that 74% of fortified fish and soy sauces comply with Cambodian regulations on iron content. 87% and 53.6% of the collected samples do not have adequate level of nitrogen and salt content, respectively. The paper will discuss additional efforts that need to be implemented to ensure the sustainability of the project, including the need to: (i) comply with International Codex; (ii) adopt mandatory legislation; and (iii) ensure enforcement.

## 1. Introduction

Iron deficiency (ID) is considered a major health problem in Cambodia given the high prevalence of anemia in children under 5 years of age and women of reproductive age, estimated to be 55.5% and 45.4% respectively in the most recent (2014) Demographic Health Survey [1]. However, iron deficiency was relatively rare among women and children with only 3% having no iron storage (ferritin concentration below 15 µg/L) [1]. In contrast, 34% of women and 48% of the children showed tissue iron deficiency, as indicated by elevated transferrin receptor concentrations above 8.3 mg/L [1]. In addition, marginal iron status was far more common, with in some regions almost half of the women having ferritin concentrations <50 µg/L [2]. This is concerning, as iron requirements during pregnancy increase considerably and iron deficiency in pregnancy is associated with pre-term delivery and low birth weight [3]. The article will primarily focus on efforts undertaken to prevent ID.

Food fortification is a widely acknowledged cost-effective solution to combat micronutrient deficiency, including ID. In essence, it is a process whereby micronutrients are added to processed foods with the aim of rapidly improving the micronutrient status of a demographic [4]. In order for fortification projects to be effective, it is advantageous to use condiments or staple foods that are widely consumed to maximize reach, with production centered on few players. Historically, various food carriers have been chosen for fortification depending on a country or region’s diet. Whilst Mexico and Ghana fortify rice [5] and salt [6], fish and soy sauces are the most logical contenders for Cambodia, as they are consumed by more than 80% of the population, including 90% for fish sauce [7].

Since 2011, the Reproductive and Child Health Alliance (RACHA) has implemented a project to fortify fish sauce and soy sauce with Sodium iron ethylenediaminetetraacetate (henceforth referred to as NaFeEDTA). The program ensures the production of fortified fish sauce for the private sector, while simultaneously supporting governmental efforts and carrying policies that make iron fortification mandatory legislation. RACHA provides technical assistance to producers and guarantees that the adequate levels of NaFeEDTA fortification are applied. In order to implement such a program, RACHA must comply with both regional and national regulations, as a consequence of Cambodia’s admission to the Association of Southeast Asian Nations (ASEAN) in 2002. In 2012, a new piece of legislation was ratified. The Senior Minister, Minister of Planning and Chairman of the National Council for Nutrition approved the proclamation (legislation: 048 NCN) which permitted the production and consumption of iron fortified fish sauces and soy sauces (IFFS/SS) [8]. This enables the use of iron fortificants in fortified fish and soy sauces, including NaFeEDTA or iron Sulphate with Citrate (FeSO_4_ + Citrate) at a level between 230 and 460 mg/L. The standard also states that total nitrogen and sodium chloride concentrations must amount to a minimum of 15 g/L and 230 g/L respectively. The recent amendments to the Codex standard for fish sauce (STAN 302-2011) detail the chemical properties required for the production of fish sauce [9]. It states that nitrogen levels have to follow AOAC 940.25, which stipulates that the minimum level of total nitrogen and sodium chloride are 10 g/L and 200 g/L respectively. Nitrogen content is commonly seen as a determinant of the quality of fish sauces in Southeast Asia and it represents an essential consumer right [10]. Therefore, the project decided to investigate the compliance of the fortified fish sauces to the international codex standards towards salt and nitrogen concentrations and to the national regulation towards iron fortification.

## 2. Methodology

The national organization RACHA collected randomly twice a year samples within the producers involved into the national fortification program. In order to maintain consistency and accuracy, the samples were sent to the Industrial Laboratory Center of Cambodia (ILCC). The ILCC provides testing services for research purposes as well as for the improvement of food product quality and safety for the regulatory departments of the Ministry of Industry, Mines and Energy (MOIME) and for non-governmental agencies (private sector-NGO) [11]. The following characteristics were analyzed: (i) total nitrogen; (ii) salt concentration; and (iii) iron concentration. All test were done in triplicates to investigate the compliance of the sauces fortified over the year: (i) iron content with the national standard for fish and soy sauce and (ii) salt and nitrogen concentrations within the codex standard for fish sauces.

The total soluble nitrogen content of fish sauce samples was measured by the Kjedahl method [12] and was expressed as g/100 mL. The measurement of the concentration of sodium chloride followed the FAO Technical Paper (AOAC 937.09) [13]. Iron content was determined after wet digestion by a flame atomic absorption spectrophotometer (model Spectr-AA-20, Varian Associates, Australia). Data entry, management and analysis were performed with Excel 2007™.

## 3. Results

Table 1 details the samples collected for this study, including where they were collected and whether they were compliant with Cambodian and international regulations. Two hundred and fifty two samples of fortified and non-fortified fish and soy sauce were collected between October 2012 and February 2014. Most of the samples were collected at the mid-term of the project in 2013. Therefore 75.8% of the samples were collected in 2013. 186 samples were collected from production plants and 66 in markets, out of which 58 iron-fortified samples do not comply with the regulations.

**Table 1:** Samples collected between October 2012 and February 2014.

	Year 2012	Year 2013	Year 2014	Total
Total Sample collected	24	191	37	252
Iron Fortified Products (IFP)—outside the standards	1	52	5	58
Sample < 10 g/L of Nitrogen	20	166	33	219
Sample < 15 g/L of Nitrogen	21	173	33	227
Sample < 200 g/L of Salt content	15	99	21	135
Sample < 230 g/L of Salt content	18	123	22	163

**Figure 1:**
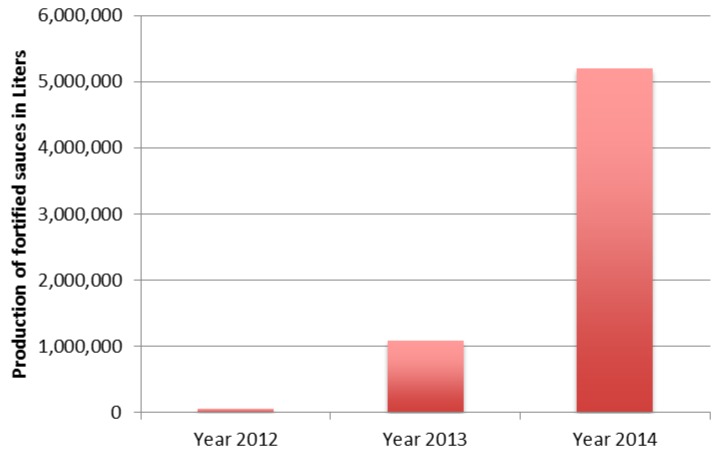
Production of iron fortified fish and soy sauce between 2012 and 2014 (in liters).

As of February 2014, RACHA recorded 42 approved private producers fortifying in 13 provinces. According to records collected by RACHA and the government (Figure 1), it is estimated that the production volume of iron-fortified fish and soy sauces between 2012 and 2014 reached 6,350,217 liters. In total, 54,500 liters of fortified fish and soy sauces were produced in 2012, compared to 1,090,810 and 5,204,907 in 2013 and 2014 respectively. Production in 2014 increased by almost five times compared to 2013. At the end of 2014, fortified products will account for almost 25% of total fish and soy sauces produced in Cambodia.

**Figure 2:**
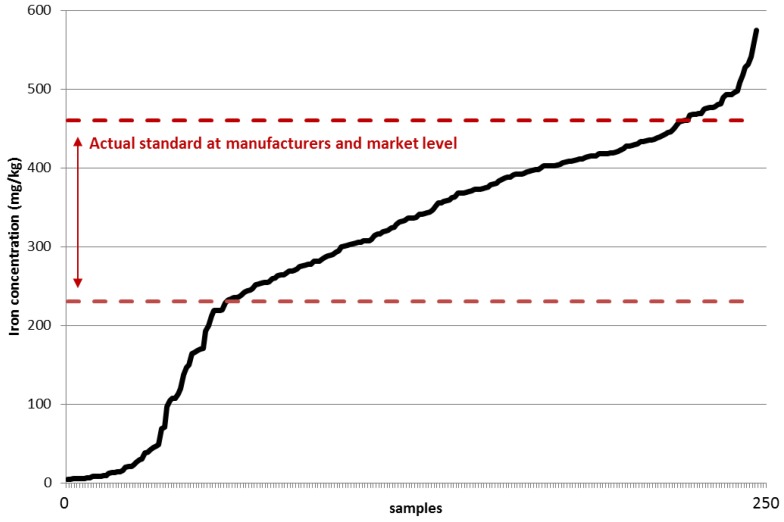
Iron values (mg/kg) in fish and soy sauces (sorted by lowest to highest iron value).

The samples highlight that producers comply, for the most part, with the fortification levels. 5.2% of fortified samples were not compliant in 2012, compared to 30.4% in 2013 and 12.9% in 2014 for an average non-compliance level of 26% for the three years. Figure 2 highlights the iron values in mg/kg in fish and soy sauces in relation to the actual standard at manufacturer and market level, which stands between 230 mg/kg and 460 mg/kg. It shows that 57 of the total samples (22.6%), fortified or unfortified, were below-standard levels of iron, compared to 29 samples (11.5%) with excessive amounts of iron levels.

**Figure 3:**
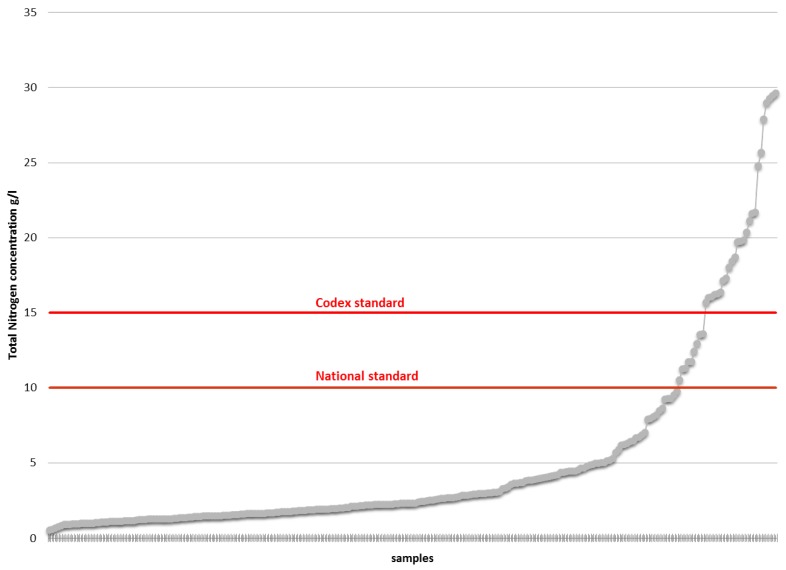
Nitrogen values (g/L) in fish sauces (sorted by lowest to highest salt value).

Figure 3 details the iron values in mg/kg in fish and soy sauces in relation to the actual standard at manufacturer and market level for year 2012, 2013 and 2014. It remains hard to draw conclusive evidence from the samples, but a few facts can be pointed out. We notice a positive trend, whereby fewer samples have lower iron content than the actual standard at manufactures and market level. 25% and 24.6% of the collected samples in 2012 and 2013, respectively, have insufficient levels of iron compared to 13.5% in 2014.

The figure also highlights that there has been an increase in fish and soy sauces with excessive amounts of iron. No samples collected in 2012 recorded any excessive amounts of iron, whereas 12.6% and 13.5% of fish and soy sauces, for 2013 and 2014 respectively had iron concentration above the actual standard at manufacturers and market level. The mean fortification level in 2012, 2013 and 2014 amount to 288.4 mg/kg, 307 mg/kg and 324.9 mg/kg respectively, totaling an average of 307.9 mg/kg for the 3 years.

The ILCC also analyzed the nitrogen and salt contents of all the samples. Findings for nitrogen are recorded in g/L. Figure 3 details the nitrogen contents of each analyzed sample and compares it to the international and Cambodian regulations, which need to contain more than 10 g/L and 15 g/L, respectively. 86.9% of the samples do not follow international standards, whilst 90% do not follow Cambodian standards.

**Figure 4:**
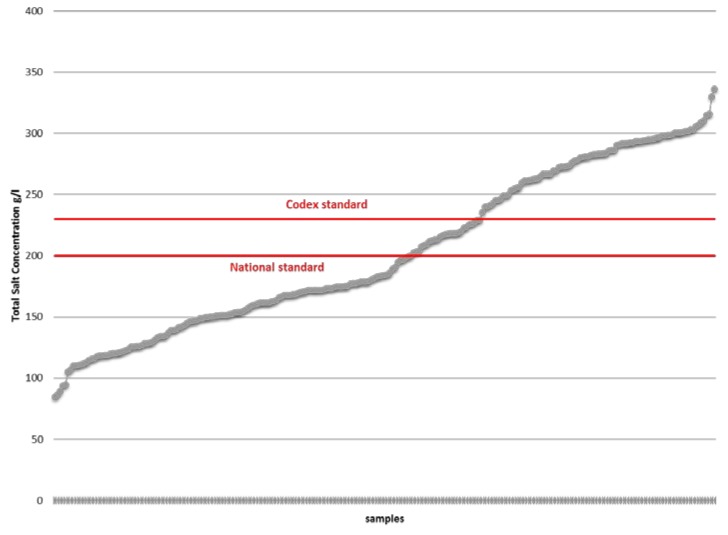
Salt values (g/L) in fish sauces (sorted by lowest to highest salt value).

Figure 4 shows the salt content and compares it to the international and Cambodian standards, which must amount to contain more than 200 g/L and 230 g/L, respectively. It shows that 53.4% of the samples do not follow international standards compared to 64.1% that do not follow Cambodian regulations.

## 4. Discussion

This study demonstrated that 74% of iron-fortified products were within the compliance levels set by Cambodian regulations. According to a daily consumption of 12 mL and the average fortification level of 307.9 mg/L, fortified sauces increase consumers’ daily iron intake by 3.69 mg. This threshold has been demonstrated as effective in combating iron deficiency among school children in Kenya [14]. The amount of iron intake via fish and soy sauces amounts to more than 12.7% of the Recommended Nutrient Intake of a woman of reproductive age [4], a considerable amount considering the insufficient rates of iron intake in Cambodia. The paper has certain limitations. The samples used for this study were primarily collected in 2013, amounting to 75.8% of total sample collected. It also does not allow for clear comparison of the iron fortification between fortified fish and soy sauces.

Several issues threaten to jeopardize the project: (i) lack of compliance with Cambodian and international regulations set by the Codex standard, (ii) fear of incompatibility between WTO trade regulation and fortification legislation, and (iii) lack of mandatory legislation and process to enforce it.

### 4.1. Lack of Compliance with Cambodian and International Regulations

Producers are at risk of being unable to sell their product as fish sauce under international regulations. 86.9% and 53.6% of the samples had inadequate nitrogen and salt content according to international regulations set in the Codex standard for fish sauce. As mentioned previously, nitrogen content is seen as a definitive quality of fish sauce in Southeast Asia and is an essential consumer right. Efforts to alter and re-adjust the chemical composition of fish sauces produced in Cambodia to the minimum levels set out in the International Codex are essential if producers want to continue selling their products. A nation-wide information program is needed to inform producers of these shortcomings. In the preparation of mandatory legislation, concerns arose in regards to World Trade Organization (WTO) trade rules.

### 4.2. Compatibility between WTO Trade Regulation and Fortification Legislation

It is essential to clearly explain the compatibility between WTO trade regulations and fortification legislation in order to ensure the success of iron-fortification projects. The principles of WTO can be found in the General Agreement on Tariffs and Trade (GATT). The agreement states that there should be no discrimination between trading partners or between imported and locally produced goods. The principles impose WTO participants to open their borders as well as to eliminate any measures favoring domestic over imported products [15]. However, these key principles do not always apply.

According to article XX (b) of the GATT, countries can adopt trade measures if they are “necessary to protect human, animal or plant life or health” [16]. Countries have to prove that the health measure is necessary, effective and that it doesn’t represent arbitrary discrimination [17]. The secondary analysis of the 2014 Cambodian Demographic and Health Survey (CDHS) [1] and a recent unpublished paper [2] record high level of women with marginal iron stores. Fortification is necessary to prevent iron deficiency and to protect human health. It has been proven as an effective nutrition intervention in the region [18].

The agreement on Technical Barriers to Trade (TBT Agreements) is probably the most relevant WTO agreement for food fortification. The TBT agreement focuses on technical regulations including product composition, labeling and packaging. According to the agreement, any domestic technical regulation must be based on existing international standards. If domestic regulations comply with international standards, the WTO assumes that no trade barrier exists [19]. For instance, a country fortifying in accordance with international regulations such as the Codex Principles for the Addition of Essential Nutrients to Foods would be acting under the TBT agreement.

The iron-fortification of fish and soy sauce in Cambodia follows the regulations set up by WTO and does not represent a trade barrier. The intervention is necessary to prevent iron deficiency and to raise fortification levels in accordance with international regulations set up by the Codex GL9-1987 [20]. To accommodate for imported products, the levels of fortification in Cambodia range from 230 to 460 mg/L. Although a harmonized regional standard has not been established, the wide range of fortification level enables products from other countries to enter the market relatively freely.

The fortification of fish and soy sauces with iron in Cambodia is a well-designed program that is compliant with the WTO and TBT agreements. It is important to note that no acclaimed fortification program established around the world has ever been challenged on trade grounds [16].

### 4.3. Facilitate the Enforcement of the Future Legislation

Once the government will have passed the mandatory legislation, the enforcement of legislation imposing fortification of fish and soy sauces with iron is necessary to scale up the project. Whilst more than 40 producers are actively fortifying, many are still reluctant to partake in the project, especially large producers. Industry partners argue that fortifying, at present, represents a competitive disadvantage. The cost of the iron-compound NaFeEDTA premix is one of main obstacles. The price of a 3.2 kg bag required to fortify 1000 L of fish or soy sauce costs $8.7. A previous study on Cambodia’s project found that many producers are not willing to fortify unless if mandatory legislation is ratified [21]. Steps to enforce mandatory legislation are required as quickly as possible to ensure that all producers fortify.

An interesting option to enable a transition into mandatory legislation is for the government to absorb parts of the additional costs of fortification, by reducing import taxes on the premix. The reduction of import taxes for a public health intervention is widely acknowledged as an effective trade measure and will not be challenged, but rather encouraged.

Simultaneously, it is vital to implement a well-designed monitoring system for quality control and assurance purposes. Fortification standards have to become fully integrated in the industry licensing, registration and production processes to assure adequate fortification.

## 5. Conclusions

Fortification is a cost-effective measure to prevent micronutrient deficiency. Cambodia’s high consumption of fish and soy sauce provides excellent vehicles for iron fortification. Promising results have been recorded since the start of the project. The study showed that the production of fortified fish and soy sauces increased from 54,500 liters in 2011 to 1,090,810 and 5,204,907 in 2012 and 2013 respectively. 76% of the collected samples comply with Cambodian regulations on iron content. It is clear that producers have to regularly monitor the content of nitrogen and salt in their products. 87% and 53.6% of the samples had inadequate nitrogen and salt content according to international regulations set in the Codex standard for fish sauce. A nation-wide information program is required to inform producers of these shortcomings. Mandatory legislation along with a well-designed monitoring system for quality control and assurance purposes needs to be enacted and enforced to build momentum on the project’s successes.
